# In Vitro and Clinical Evaluation of the Anti-Wrinkle Efficacy of Medipep-6PN, a Novel Peptide Identified by Phage Display

**DOI:** 10.3390/ijms27041753

**Published:** 2026-02-11

**Authors:** Jinho Bang, Kyuhyuk Im, Yul-Lye Hwang, Mi Yoon Kim, Jae Nam Yun, Min Youl Chang, Sunghyun Kim, Jeung-hoon Lee

**Affiliations:** 1 SKINMED R&D Center, Daejeon 34037, Republic of Korea; bjh619@skinmed.biz (J.B.);; 2 College of Pharmacy, Chungbuk National University, Cheongju 28160, Republic of Korea; 3 Bio-Healthcare Materials Center, Korea Institute of Ceramic Engineering and Technology, Cheongju 28160, Republic of Korea; 4 SKINMED Clinical Trials Center, Daejeon 34050, Republic of Korea

**Keywords:** phage display, muscle-type nicotinic acetylcholine receptor, matrix metalloproteinase-1, collagen, peptide, anti-wrinkle, clinical study

## Abstract

Face wrinkles caused by skin aging can be classified into dynamic wrinkles, which are caused by repetitive contraction of facial expression muscles, and static wrinkles, which are related to extracellular matrix damage and collagen breakdown caused by ultraviolet and oxidative stress. These two mechanisms are closely related, and prolonged, repetitive muscle contractions act as mechanical stress that promotes extracellular matrix degradation within the dermis, accelerating wrinkle formation. In this study, we used phage display to develop a novel peptide, Medipep-6PN, that targets both muscle-type nicotinic acetylcholine receptor (muscle nAChR), a major cause of dynamic wrinkles, and matrix metalloproteinase-1 (MMP-1), a cause of static wrinkles. In this study, the kinetic analysis of Medipep-6PN using surface plasmon resonance analysis showed that the equilibrium dissociation constant (*K_D_*) for muscle nAChR α1 was 9.56 × 10^−6^ M, and the *K_D_* for MMP-1 was 1.25 × 10^−6^ M. Calcium imaging analysis in TE671 cells expressing the muscle nAChR pentamer determined that Medipep-6PN inhibited muscle nAChR channel activity in a concentration-dependent manner, and in particular, it was confirmed that about 80% of muscle nAChR channel activity was inhibited under 30 μM of Medipep-6PN. In addition, in an in vitro test performed to evaluate MMP-1 activity, Medipep-6PN inhibited MMP-1 activity in a concentration-dependent manner, and the IC_50_ was 4.2 ppm. When measuring MMP-1 gene expression in UVB-induced human fibroblasts, 1 ppm of Medipep-6PN showed a 52.3% decrease compared to UVB irradiation alone. When measuring type I procollagen synthesis in human fibroblasts, Medipep-6PN increased procollagen Iα1 production in a concentration-dependent manner, and concentrations between 5 and 10 ppm of Medipep-6PN significantly increased collagen I production. No significant toxicity was observed in cytotoxicity tests, demonstrating its safety. Furthermore, in a clinical study evaluating wrinkle improvement efficacy in 25 adults over a four-week period, the Medipep-6PN group demonstrated statistically significant reductions in wrinkle depth (by 10.16%) and wrinkle volume (by 13.00%), demonstrating efficacy comparable to that of commercially available functional anti-wrinkle ingredients. In conclusion, this study demonstrates that Medipep-6PN, developed to target two mechanisms—the relaxation of muscle contraction and the inhibition of collagen degradation—is a functional peptide effective in improving skin wrinkles, confirmed through in vitro evaluation and clinical studies.

## 1. Introduction

Skin aging is caused by complex interactions between intrinsic (chronological) and extrinsic (environmental) factors and leads to multifaceted changes such as wrinkle formation, pigmentation, and loss of elasticity [1,2,3,4,5]. Among these visible signs of aging, wrinkles are classified into dynamic and static wrinkles according to their mechanisms of formation [6]. Dynamic wrinkles are formed when the neurotransmitter acetylcholine (ACh) binds to muscle-type nicotinic acetylcholine receptors (muscle nAChRs) and induces muscle contraction [7,8]. In contrast, static wrinkles are formed by factors such as ultraviolet (UV) exposure and oxidative stress, which induce collagen degradation through the production of matrix metalloproteinases (MMPs), which in turn degrade the extracellular matrix (ECM), including collagen fibers [9,10,11]. These mechanisms do not act independently; in particular, prolonged, repetitive muscle contraction acts as mechanical stress, accelerating ECM degradation and wrinkle formation [12,13].

Currently, the standard treatment for wrinkle improvement is botulinum toxin injection therapy. Botulinum toxin (BoNT) binds with high affinity to peripheral cholinergic nerve terminals, enters the cytoplasm, and cleaves SNARE proteins, thereby blocking neurotransmitter release and inhibiting muscle contraction, which is the cause of dynamic wrinkles, thus inducing an anti-wrinkle effect [14,15,16]. However, the burden of repeated BoNT injections on patients and the potential side effects and safety concerns associated with the use of neurotoxins have led to a persistent need for non-invasive alternatives, such as functional cosmetic ingredients, with minimized concerns about side effects [17,18,19,20]. Therefore, functional cosmetic ingredients with anti-wrinkle effects through inhibition of muscle contraction or collagen degradation and synthesis have been developed as non-invasive alternatives. Representative examples include Argireline^®^ (Lipotec, Spain), which inhibits the formation of the SNARE complex and thus inhibits the release of the neurotransmitter ACh, thereby exhibiting an anti-wrinkle effect. SYN^®^-AKE (DSM, Switzerland) acts on muscle nAChR to block muscle contraction signal transmission, thereby exhibiting an anti-wrinkle effect. Furthermore, Matrixyl^®^ (Sederma, France) exhibits an anti-wrinkle effect by increasing collagen I synthesis in fibroblasts. These commercially available cosmetic ingredients are single-target ingredients developed to target only one of either dynamic wrinkles (muscle contraction) or static wrinkles (collagen degradation/synthesis) [21,22,23,24,25,26]. However, dynamic and static wrinkle mechanisms are interconnected; specifically, muscle contractions accelerate ECM degradation. Consequently, existing single-target ingredients cannot fully block the interplay between dynamic and static wrinkles, which may limit their overall clinical efficacy. In contrast, an agent that simultaneously targets muscle contraction (nAChR) and enzymatic degradation (MMP-1) is expected to completely block this interaction, providing significant clinical anti-wrinkle improvement.

In this study, we focused on this aspect and, to enhance anti-wrinkle efficacy, used a phage display approach to discover novel peptides that target both muscle nAChR and MMP-1, key mediators of dynamic and static wrinkles. Phage display technology is a technique that enables the expression of a desired protein (e.g., antibody or peptide) on the surface proteins of bacteriophages, allowing that protein to be displayed on the phage surface [27,28,29,30]. Biopanning is a method used to select phages that bind strongly to specific target substances (antigens, ligands, etc.) from a phage display library through repeated cycles of washing and elution [29,30]. Through this biopanning process, phages with high binding affinity for the target materials are ultimately selected.

In our previous study, we constructed a phage display library of linear random 6-mer peptides using NNK codon-based randomization, securing a library of approximately 1.23 × 10^7^ diverse clones [31]. In this study, we identified dual-binding hexapeptides targeting two proteins (muscle nAChR α1 and MMP-1) through a sequential biopanning strategy using this library. Based on the selected lead sequence, Arg-Lys-Trp-Arg-Tyr-Arg (RKWRYR), we developed Medipep-6PN, a palmitoyl-hexapeptide, by substituting a palmitoyl group at the N-terminus to enhance skin permeability and in vivo stability. The palmitoyl group is a well-known functional group that enhances the lipid affinity of peptides, strengthens the interaction with stratum corneum lipids, improves skin permeability, confers resistance to proteolytic enzymes, and increases in vivo stability [32,33,34].

The aim of this study was to elucidate the molecular mechanism of a novel palmitoyl-hexapeptide, Medipep-6PN, discovered using the phage display method, to determine the in vitro toxicity and anti-aging effects, and to demonstrate its safety and efficacy through human clinical studies, thereby suggesting its potential as a functional cosmetic ingredient for improving wrinkles.

## 2. Results

### 2.1. Screening of Candidate Peptides by Biopanning

**Figure 1:**
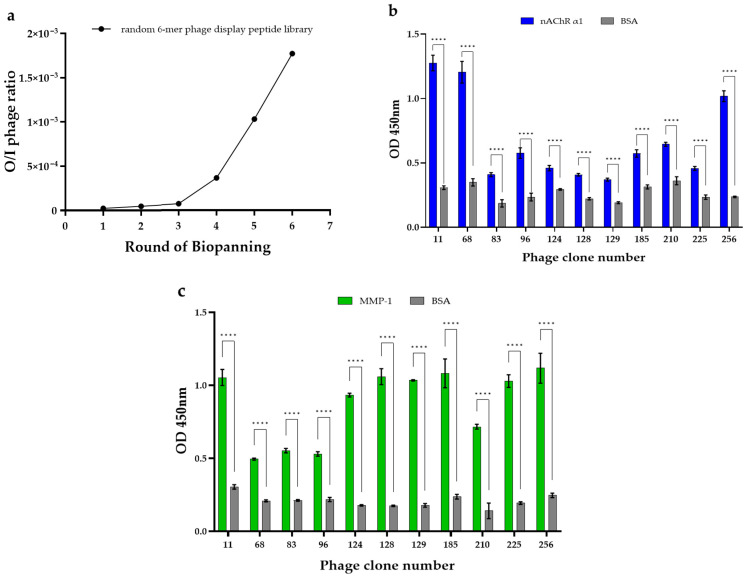
Biopanning and enzyme-linked immunosorbent assay (ELISA) results. (**a**) The output/input (O/I) phage ratio of the random 6-mer phage display peptide library in each round of biopanning against muscle nAChR α1 and MMP-1. (**b**) OD values of phage clones binding to muscle nAChR α1 compared with the negative control BSA. (**c**) OD values of phage clones binding to MMP-1 compared with BSA. Data are expressed as the mean ± SEM of three independent biological experiments, each performed in technical triplicate. Statistical significance was determined by two-way ANOVA followed by Sidak’s multiple comparisons test (target vs. BSA within each clone). **** *p* < 0.0001 vs. BSA.

To discover peptides that specifically bind to muscle nAChR α1 and MMP-1 target proteins, we performed biopanning against the two target proteins using a random 6-mer peptide library constructed using the phage display method. Biopanning was performed over six rounds, and the output/input (O/I) phage ratio was calculated for each round of biopanning. As biopanning rounds progressed, the O/I ratio of phages gradually increased (Figure 1a), and the O/I phage ratio in the sixth round was about 75 times higher than that of the first round (Table 1). This increase in the O/I phage ratio shows that phages binding to muscle nAChR α1 and MMP-1 were gradually enriched during biopanning.

To select phage clones specifically bound to both target proteins from the enriched phage pool, 270 individual phage clones were randomly chosen from the phage population recovered after the sixth round, and their binding signals to each target protein were evaluated by using an enzyme-linked immunosorbent assay (ELISA). As a result, among these 270 clones, 11 phage clones that exhibited the highest binding signals (OD values) to both target proteins relative to the negative control bovine serum albumin (BSA) were selected (Figure 1b,c). The DNA inserts of these clones were sequenced to determine the amino acid sequences of the corresponding 6-mer peptides (Table 2 and Table S1), and based on these sequences, 11 candidate peptides were synthesized via solid-phase peptide synthesis (SPPS) (Table A1).

### 2.2. Selection of the Final Peptide by Surface Plasmon Resonance

**Figure 2:**
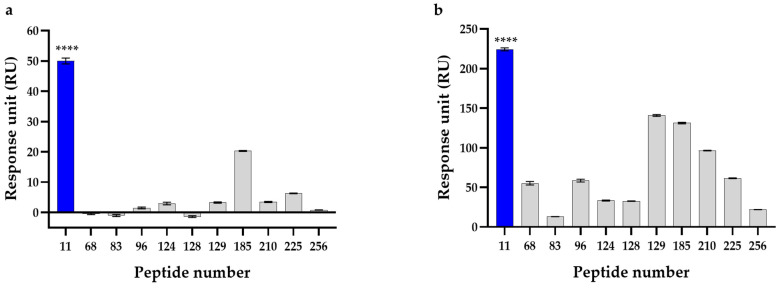
Response unit (RU) values of 11 candidate peptides measured by SPR. (**a**) RU values of interaction between 11 candidate peptides and muscle nAChR α1. (**b**) RU values of interaction between 11 candidate peptides and MMP-1. Data are shown as mean ± SEM of three independent biological experiments, each performed in technical triplicate. Statistical significance was determined by one-way ANOVA followed by Tukey’s multiple comparisons test. **** *p* < 0.0001 compared to all other tested peptides.

Surface Plasmon Resonance (SPR) is a widely used technique for characterizing peptide–protein interactions, enabling the real-time monitoring of binding and dissociation kinetics between immobilized proteins and soluble analytes [35,36,37,38]. In this study, SPR was used to analyze the interactions between muscle nAChR α1 and MMP-1 and 11 candidate peptides, and to compare their relative binding responses. First, muscle nAChR α1 was immobilized on a sensor chip, and the change in a response unit (RU) that occurs when a peptide binds to muscle nAChR α1 was measured in real time while each peptide was injected at a concentration of 100 μM. As a result, Peptide 11 showed the highest response of about 50 RU, indicating that the binding response to muscle nAChR α1 was the strongest among the 11 candidate peptides (Figure 2a). Next, using the same peptides, the binding responses to MMP-1 were evaluated. MMP-1 was immobilized on a sensor chip, and RUs were measured under the same conditions. Peptide 11 again showed the highest response, with a value of approximately 227 RUs, which was higher than that of any other peptide (Figure 2b). Based on these results, Peptide 11, which showed the highest binding responses to both muscle nAChR α1 and MMP-1, was selected as the final product. Furthermore, to improve skin permeability and in vivo stability, palmitoyl-hexapeptide was developed by substituting a palmitoyl group at the N-terminus of Peptide 11 and was named Medipep-6PN.

### 2.3. Evaluation of the Influence of Palmitoylation on Peptides by Using In Silico Docking Molecular Modeling

**Figure 3:**
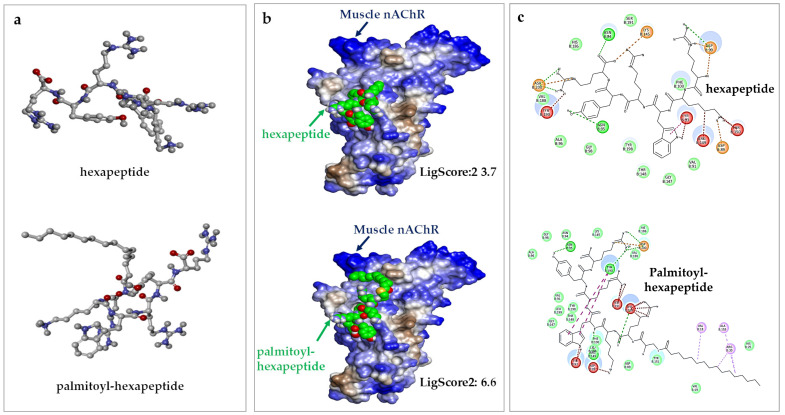
Predicted in silico molecular docking modeling. (**a**) Molecular structure of hexapeptide and palmitoyl-hexapeptide. (**b**) 3D docking models of hexapeptide (**top**) and palmitoyl-hexapeptide (**bottom**) with muscle nAChR. LigScore2 of hexapeptide (**top**) is 3.7, and Ligscore2 of palmitoyl-hexapeptide (**bottom**) is 6.6. (**c**) Visualization of molecular dynamics-based intermolecular interactions with Ligplot.

To evaluate the potential influence of palmitoylation on the receptor-binding properties of the peptides, in silico docking molecular modeling was performed to predict the binding modes of hexapeptide and palmitoyl-hexapeptide. The analysis predicted that both peptides could potentially form binding poses near loop C at the interface of the α1 and ε subunits of muscle nAChR (Figure S1). The predicted binding potential, represented by LigScore2, was calculated to be 6.6 for palmitoyl-hexapeptide, showing a relatively higher predictive score compared to hexapeptide (3.7) (Figure 3b). Furthermore, visualization of the predicted intermolecular interactions using Ligplot suggested that palmitoyl-hexapeptide might form a higher frequency of hydrogen bonds and hydrophobic interactions with the receptor than hexapeptide (Figure 3c).

### 2.4. Analysis of the Binding Affinity by Surface Plasmon Resonance

**Figure 4:**
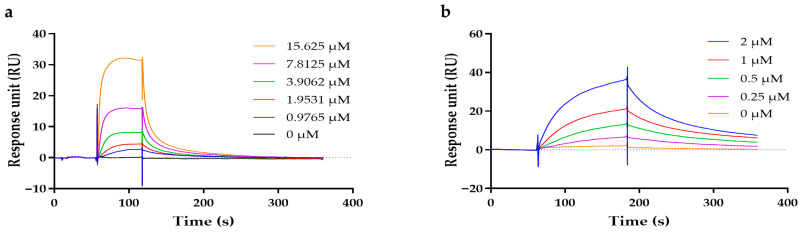
Kinetic analysis of the binding affinity of palmitoyl-hexapeptide to muscle nAChR α1 and MMP-1. (**a**) Sensorgram for binding affinity of muscle nAChR α1 to palmitoyl-hexapeptide. (**b**) Sensorgram for binding affinity of MMP-1 to palmitoyl-hexapeptide.

The binding affinity between ligands (hexapeptide and palmitoyl-hexapeptide) and proteins (muscle nAChR and MMP-1) was measured using SPR. First, muscle nAChR α1 was immobilized on a sensor chip, and the change in a RU that occurs when hexapeptide and palmitoyl-hexapeptide bind to muscle nAChR α1 was measured in real time while each peptide was injected at various concentrations (Figure 4a and Figure S2). Kinetic analysis showed that palmitoyl-hexapeptide had an association rate constant (*k_a_*) of 5.38 × 10^3^ M^−1^ s^−1^ and a dissociation rate constant (*k_d_*) of 5.14 × 10^−2^ s^−1^, yielding an equilibrium dissociation constant (*K_D_*) of 9.56 × 10^−6^ M (Table 3). In contrast, the *K_D_* of hexapeptide for muscle nAChR α1 was 2.52 × 10^−3^ M. Thus, palmitoyl-hexapeptide exhibited an approximately 250-fold higher binding affinity for muscle nAChR α1 than hexapeptide, which was consistent with the trend predicted by the in silico docking.

Next, MMP-1 was immobilized on a sensor chip, and the change in a RU that occurs when hexapeptide and palmitoyl-hexapeptide bind to MMP-1 was measured in real time while each peptide was injected at various concentrations (Figure 4b and Figure S3). For hexapeptide, the association and dissociation rate constants were 1.06 × 10^3^ M^−1^ s^−1^ and 6.66 × 10^−3^ s^−1^, respectively, resulting in a *K_D_* of 6.24 × 10^−6^ M. For palmitoyl-hexapeptide, the corresponding *k_a_* and *k_d_* values were 7.70 × 10^3^ M^−1^ s^−1^ and 9.62 × 10^−3^ s^−1^, yielding a *K_D_* of 1.25 × 10^−6^ M (Table 3). Therefore, palmitoyl-hexapeptide also showed approximately five-fold higher binding affinity for MMP-1 than hexapeptide.

### 2.5. Evaluation of Muscle nAChR Channel Activity by Ratiometric Fluorescence Imaging

**Figure 5:**
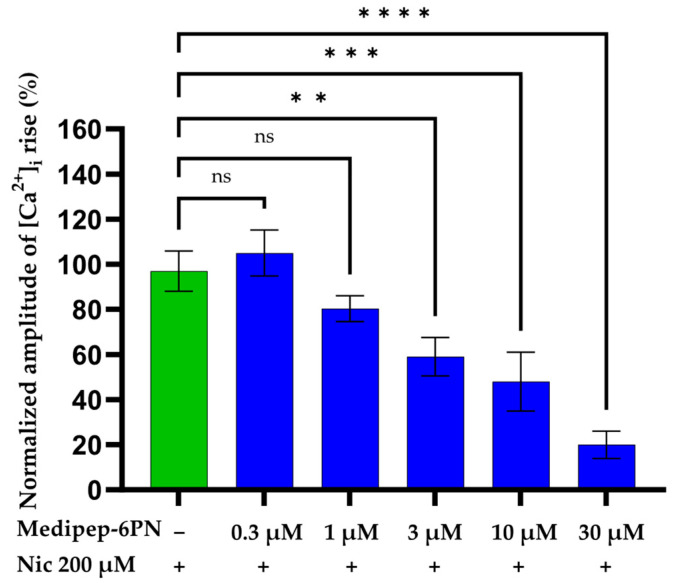
Inhibitory effect of Medipep-6PN on muscle nAChR channel activity. TE671 cells were pretreated with 0.3 to 30 μM Medipep-6PN and then stimulated with nicotine (blue bars). Intracellular calcium ion ([Ca^2+^]_i_) influx in nicotine-treated group (200 μM, green bars) was assumed to be 100%, and changes in [Ca^2+^]_i_ concentration were compared between groups. Data are presented as mean ± standard error of mean of three independent biological experiments, each performed in technical triplicate. Statistical significance was determined by one-way ANOVA followed by Tukey’s multiple comparisons test. ** *p* < 0.01, *** *p* < 0.001, **** *p* < 0.0001 vs. the nicotine-treated control group; ns, not significant.

The influx of calcium ions through muscle nAChR channels at various concentrations of Medipep-6PN was measured using ratiometric fluorescence imaging. TE671 cells expressing muscle nAChRs were treated with Medipep-6PN at concentrations of 0.3 to 30 μM, followed by 200 μM nicotine. Intracellular calcium ion ([Ca^2+^]_i_) influx was measured using calcium imaging. Compared to the nicotine-only control group, the Medipep-6PN-treated groups showed a concentration-dependent decrease in [Ca^2+^]_i_ influx, with a decrease of approximately 80% in the Medipep-6PN 30 μM-treated group (Figure 5). These results indicate that Medipep-6PN binds to muscle nAChRs in TE671 cells and inhibits nicotine-induced channel activation in a concentration-dependent manner. This suggests that Medipep-6PN acts as a ligand that binds to muscle nAChRs, thereby inhibiting channel activity.

### 2.6. In Vitro Efficacy on Inhibition of MMP-1 Activity and Collagen Biosynthesis

The inhibitory effect of Medipep-6PN on MMP-1 activity was evaluated using a fluorogenic assay. We applied Medipep-6PN at a concentration of 1 to 100 ppm using a commercial EnzCheck Gelatinase/Collagenase Assay Kit. As a result, Medipep-6PN showed a concentration-dependent rate of MMP-1 activity inhibition. The inhibition rates of Medipep-6PN were 22.1% at 1 ppm, 38.9% at 5 ppm, 65.0% at 10 ppm, and 80% or more at 50 ppm and 100 ppm. Therefore, it was confirmed that the IC_50_ of Medipep-6PN was 4.2 ppm.

**Figure 6:**
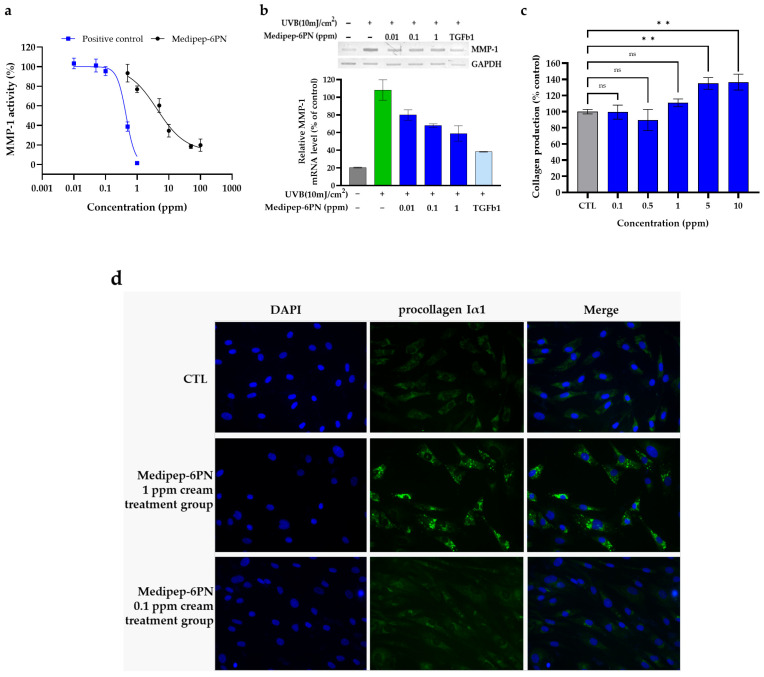
In vitro evaluation of the effects of Medipep-6PN on MMP-1 activity, MMP-1 expression, and collagen I synthesis. (**a**) Analysis of the inhibitory effect of Medipep-6PN on MMP-1 activity using a fluorescence assay performed in technical triplicate. The inhibition rate is expressed as a percentage compared to the untreated control group, and 1,10-phenanthroline monohydrate served as a positive control. (**b**) Evaluation of the effects of Medipep-6PN on MMP-1 mRNA expression in HDFs induced by UVB (10 mJ/cm^2^) irradiation, performed in technical triplicate. Gray bars (non-irradiated normal control group), green bars (UVB only), blue bars (Medipep-6PN-treated group), and light blue bars (TGF-β1-treated group) are the positive control. (**c**) The results of the stimulation of collagen I production by Medipep-6PN in HDFs, performed in technical triplicate. The production rate is expressed as a percentage compared to the untreated control group, and statistical significance was determined by one-way ANOVA followed by Tukey’s multiple comparisons test. ** *p* < 0.01 vs. the untreated control (CTL) group; ns, not significant. (**d**) Immunofluorescence images demonstrating the effects of Medipep-6PN on collagen I production in HDFs. First column: DAPI (blue); second column: procollagen Iα1 (green); third column: merged images of nuclei and procollagen I.

The effect of Medipep-6PN on MMP-1 gene expression in ultraviolet B (UVB)-induced human dermal fibroblasts (HDFs) was evaluated using RT-PCR. HDFs were first irradiated with UVB (10 mJ/cm^2^) and then treated with 0.01, 0.1, or 1 ppm of Medipep-6PN, followed by RT-PCR analysis. As a result, the level of MMP-1 mRNA expression in the negative control group (UVB only) increased approximately 4.5-fold compared to the normal control group (non-irradiated), and the 1 ppm Medipep-6PN treatment group showed a 52.3% decrease in MMP-1 mRNA expression compared to the negative control group. These results demonstrate that Medipep-6PN significantly inhibits UVB-induced MMP-1 gene expression even at a low concentration of 1 ppm (Figure 6b).

To determine the effect of Medipep-6PN on type I collagen biosynthesis, HDFs were treated with Medipep-6PN at concentrations of 0.1, 0.5, 1, 5, and 10 ppm, and then the levels of secreted procollagen Iα1 in the culture supernatant were quantified by ELISA. As a result, compared with the negative control (vehicle), procollagen Iα1 levels increased by 10.9% at 1 ppm, 34.9% at 5 ppm, and 36.6% at 10 ppm, and collagen I production was significantly increased at concentrations between 5 and 10 ppm (Figure 6c). The effect of Medipep-6PN on collagen I synthesis was confirmed by immunofluorescence staining. HDFs were treated with Medipep-6PN cream at 0.1 ppm or 1 ppm for 24 h, and intracellular collagen I expression was visualized by immunofluorescence. In the group treated with 1 ppm Medipep-6PN cream, procollagen Iα1 fluorescence intensity increased by approximately 42% compared with the vehicle control (Figure 6d). These results suggest that Medipep-6PN stimulates type I collagen biosynthesis.

### 2.7. Evaluation of Cytotoxicity

**Figure 7:**
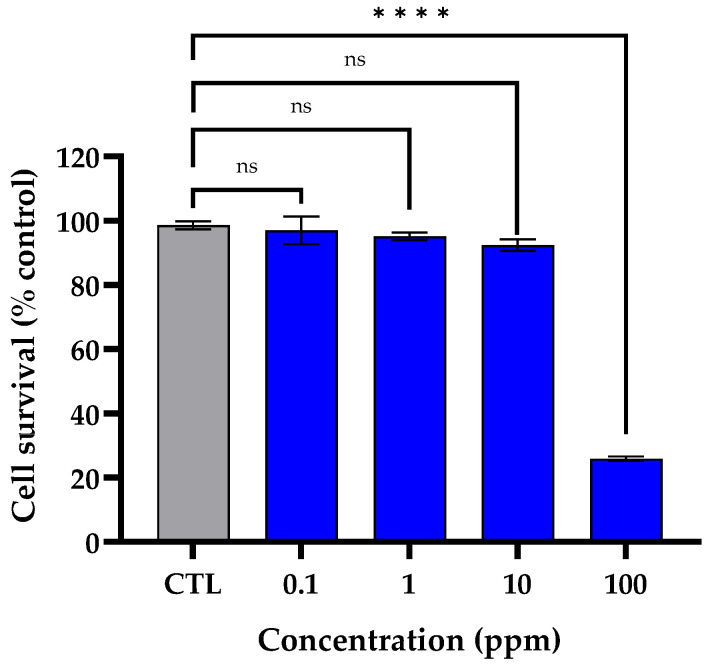
Cytotoxicity of Medipep-6PN in HDFs. HDFs were treated with Medipep-6PN at 0.1, 1, 10, or 100 ppm, and cell viability was assessed by using an MTT assay after 24 h. Data are expressed as the mean ± SEM of three independent biological experiments, each performed in technical triplicate. Statistical significance was determined by one-way ANOVA followed by Tukey’s multiple comparisons test. **** *p* < 0.0001 vs. the untreated control (CTL) group; ns, not significant.

An MTT assay was performed to evaluate the cytotoxicity of Medipep-6PN in HDFs. After treating HDFs with Medipep-6PN at concentrations of 0.1, 1, 10, and 100 ppm, cell viability was measured using MTT. At concentrations of 0.1 to 10 ppm, cell viability was maintained at over 90%, indicating no toxicity. However, at 100 ppm, cell viability significantly decreased to less than 30% compared to the control group (Figure 7). Therefore, the maximum concentration without cytotoxicity in this test was 10 ppm.

### 2.8. Clinical Study: Evaluation of Wrinkle Improvement Efficacy

**Figure 8:**
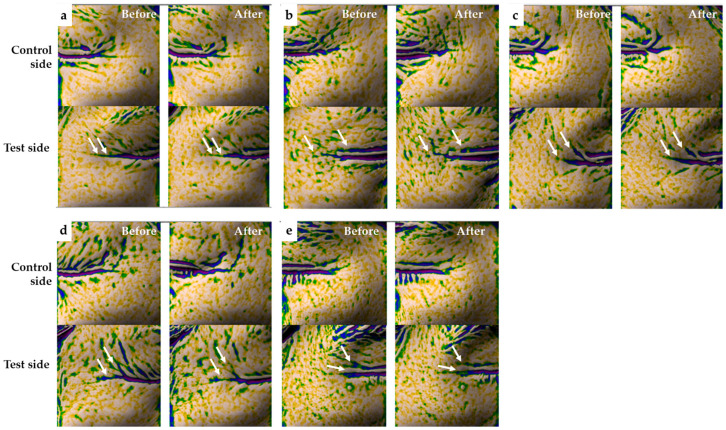
Antera 3D^®^ results images before and after application of each substance to evaluate improvement in crow’s feet wrinkles. (**a**) Representative Antera 3D^®^ images of crow’s feet wrinkles taken before and after 4 weeks of treatment in control (placebo) and test groups (Medipep-6PN). White arrows indicate areas where wrinkle improvement was observed in test group. (**b**–**e**) Changes in wrinkle depth and volume by comparative ingredients: (**b**) SYN^®^-AKE, (**c**) Medimin A, (**d**) retinol, and (**e**) retinyl palmitate.

After 4 weeks of application, the Medipep-6PN treatment group showed significant improvement in crow’s feet wrinkle parameters on the test side compared to baseline (Table 4). Specifically, wrinkle depth and volume on the test side decreased by 10.16% and 13.00%, respectively, whereas minimal changes were observed on the placebo (control) side during the same period (Table 4). In the intra-individual comparison, the Medipep-6PN test side exhibited a significant reduction in both wrinkle depth and volume compared to the control side (Table 4). Furthermore, visual comparison of representative Antera 3D^®^ images confirmed a noticeable alleviation of crow’s feet wrinkle contours after 4 weeks (Figure 8a). The comparative active ingredient groups (SYN^®^-AKE, Medimin A, retinol, and retinyl palmitate) also showed significant reductions in wrinkle depth and volume on the test side compared to the control side after 4 weeks (Table 4). The magnitude of improvement observed with these functional ingredients was generally comparable to that of the Medipep-6PN group (Figure 8b–e; Table 4). Regarding safety evaluation, no adverse skin reactions such as erythema, itching, stinging, or burning sensation were reported during the 4-week application period, with all subjects recorded as “No symptoms” (Table S2).

## 3. Discussion

Facial wrinkles, one of the most prominent features of skin aging, can be classified into dynamic wrinkles, which are formed by repetitive contraction of facial expression muscles, and static wrinkles, which are caused by structural damage to the ECM due to collagen degradation caused by matrix metalloproteinases, such as UV exposure and oxidative stress [1,2,3,4,5,6]. In particular, prolonged, repetitive muscle contractions can accelerate wrinkle formation due to ECM degradation by acting as mechanical stress [12,13].

In this study, we aimed to develop a peptide with enhanced wrinkle-improving effects by targeting both muscle nAChR and MMP-1, mediators of both dynamic and static wrinkles, using phage display. In a previous study, we reported a peptide with anti-wrinkle effects targeting muscle nAChR using phage display [31]. Using a peptide library acquired in a previous study, we sequentially performed biopanning to target muscle nAChR, a mediator of dynamic wrinkles, and MMP-1, a mediator of static wrinkles, to secure a novel peptide library. Furthermore, after screening 11 peptides with high binding affinity to muscle nAChR α1 and MMP-1 through a six-round biopanning process, we selected a hexapeptide (RKWRYR) with the highest binding affinity for muscle nAChR α1 and MMP-1 using SPR analysis. To improve skin permeability and in vivo stability, we substituted a palmitoyl group at the N-terminus to develop palmitoyl-hexapeptide [33,34,35,39].

The in silico docking results of this study provide a structural hypothesis that palmitoylation may enhance the structural stability of the binding site by increasing hydrogen bonding and hydrophobic interactions with muscle nAChR α1. Specifically, the high LigScore2 value of palmitoyl-hexapeptide suggests that the lipid tail anchors into the hydrophobic pocket of the receptor, thereby potentially increasing binding affinity. However, molecular docking simulations are inherently predictive and should be interpreted as hypothesis-generating tools rather than confirmatory evidence. In this study, while LigScore2 suggests enhanced affinity, we did not perform extensive statistical validations such as RMSD clustering or experimental binding assays (e.g., site-directed mutagenesis). Thus, these computational insights warrant further verification through structural biology approaches [40,41]. To support these predictive models, we performed SPR analysis to measure the actual binding affinity between biomolecules. The results showed that palmitoyl-hexapeptide exhibited equilibrium dissociation constants (*K_D_*) of 9.6 μM for muscle nAChR α1 and 1.2 μM for MMP-1. These data demonstrate that palmitoylation substantially increases the physical binding affinity of the peptide to its target proteins, aligning with the enhancement trends predicted by the docking models. In conclusion, the complementary results from the docking simulations and SPR experimental data provide a robust scientific basis for the theory that palmitoylation enhances both the binding affinity and the in vivo stability of the peptide for its target proteins. Future research on the development of cosmetics using Medipep-6PN will assess the effects of palmitoylation on skin permeability and interactions with stratum corneum lipids. The effects of palmitoylation on transdermal absorption and metabolic stability will be assessed using Franz diffusion cell skin permeation experiments and HPLC quantitative analysis of the residual amount of Medipep-6PN in the stratum corneum [32,42,43].

In this study, we predicted that Medipep-6PN would bind near the C loop of muscle nAChR α1 through molecular modeling using computer simulations. Furthermore, in vitro experiments were performed on TE671 cells expressing muscle nAChRs. After pretreatment with various concentrations of Medipep-6PN, nicotine was added to measure intracellular calcium concentration (fluorescence intensity) and assess the degree of muscle nAChR channel activation. As a result, Medipep-6PN concentration-dependently decreased intracellular calcium fluorescence intensity, suggesting that it inhibits muscle nAChR channel activation. These results suggest that Medipep-6PN binds to muscle nAChRs and inhibits muscle nAChR channel activity, thereby reducing intracellular calcium ion influx, blocking signal transduction, and suppressing muscle contraction, resulting in its anti-wrinkle effect. This is consistent with previous studies demonstrating that muscle nAChR-targeting peptides exert anti-wrinkle effects by modulating neuromuscular signaling [22,28,31,44,45,46,47].

However, in this study, the IC_50_ value at which Medipep-6PN inhibits muscle nAChR channel activity could not be determined. In a follow-up study, we plan to use electrophysiological methods such as whole-cell patch clamp recordings to measure the IC_50_, a quantitative indicator of the wrinkle-improving effect of Medipep-6PN which acts by binding to muscle nAChRs and inhibiting muscle contraction. These results will be compared and evaluated with mechanistic studies [45,48]. In addition, we plan to establish an in vitro skeletal muscle cell contraction model using differentiated mouse skeletal muscle cells and quantitatively evaluate the effect of Medipep-6PN on the contraction–relaxation dynamics of muscle fibers to investigate the correlation with the wrinkle-improving effect in humans [49,50,51,52].

MMP-1 is a representative collagen-degrading enzyme and a mediator of static wrinkle formation caused by photoaging and oxidative stress [9,10,11]. In this study, we confirmed that Medipep-6PN concentration-dependently inhibited MMP-1 enzyme activity with an IC_50_ value of 4.2 ppm through an in vitro MMP-1 activity assay. These results scientifically demonstrate that Medipep-6PN, developed to target the collagen-degrading enzyme MMP-1, exhibits an anti-wrinkle effect by suppressing MMP-1 activity and mRNA gene expression. The evaluation method for anti-wrinkle efficacy is based on the in vitro MMP-1 activity inhibition and UVB-induced MMP-1 mRNA expression assay recommended by the Ministry of Food and Drug Safety (MFDS) guidelines [53]. In future follow-up studies, we plan to predict the molecular structure, intermolecular interactions, and binding sites of Medipep-6PN and MMP-1 through docking molecular modeling using computer simulations and to elucidate the correlation with in vitro efficacy evaluations [38].

While our findings demonstrate that Medipep-6PN effectively inhibits muscle nAChR activation and suppresses MMP-1 enzymatic activity, further investigation is required to fully elucidate its target binding specificity. Muscle nAChRs are hetero pentamers containing the α1 subunit, whereas neuronal subtypes are composed of different subunits (e.g., α7, α4β2), which share homologous ligand-binding domains. Although our docking simulation predicts that Medipep-6PN interacts with the loop C region at the α1-ε interface—a site distinct from neuronal subtypes—experimental validation using subtype-selective antagonists or comparative functional assays against neuronal nAChRs is necessary to rule out potential cross-reactivity [54]. Similarly, given the high structural conservation of the catalytic domain across the MMP family, the observed MMP-1 inhibition (IC_50_ = 4.2 ppm) does not inherently guarantee selectivity over other isoforms such as MMP-2 or MMP-9. Future studies will therefore focus on selectivity profiling against a broader panel of nAChR subtypes and MMP isoforms, as well as competitive binding assays, to confirm the precise mode of action and exclude off-target effects [55,56].

Medipep-6PN significantly increased collagen production in HDFs, and immunofluorescence imaging also showed a concentration-dependent increase in procollagen Iα1 fluorescence intensity. These results indicate that Medipep-6PN increases collagen I biosynthesis. Although collagen I biosynthesis was not the target during the phage display screening phase, these results suggest that the palmitoyl group of Medipep-6PN may have contributed to collagen I biosynthesis by promoting electrostatic and hydrophobic interactions with membrane phospholipids or receptor-binding sites. Future studies will aim to elucidate the mechanism of collagen I biosynthesis by Medipep-6PN [57,58].

Our human application study demonstrated that topical application of Medipep-6PN for 4 weeks significantly reduced the depth and volume of crow’s feet wrinkles on the test side by 10.16% and 13.00%, respectively, compared to baseline (*p* < 0.05). These improvements were comparable to those observed with established functional ingredients such as SYN^®^-AKE and retinol. Furthermore, no adverse skin reactions were reported during the study period, confirming the excellent skin tolerability of Medipep-6PN. However, this study has several limitations inherent to its nature as a pilot clinical trial. First, the small sample size of five subjects per group (total *n* = 25) may limit the statistical power of the analysis. Although statistically significant improvements in wrinkles were observed, the restricted sample size could constrain the statistical robustness and generalizability of the findings; thus, validation in a larger sample size is required to confirm reproducibility. Second, the wide age range of participants (30–65 years) introduces potential age-related variability in skin aging conditions. While we employed a split-face design to minimize inter-individual biological variation and control for treatment effects within the same subject, the lack of stratified analysis by age group remains a limitation of this study.

Therefore, the current results should be regarded as promising preliminary evidence of the wrinkle-improving potential of Medipep-6PN. Definitive establishment of its efficacy will require follow-up randomized, double-blind, placebo-controlled trials (RCTs) with an expanded sample size.

## 4. Materials and Methods

### 4.1. Materials and Reagents

Recombinant human AChR subunit α1 (recombinant human AChR subunit α1, Cat. No. MBS963713) used in this study was purchased from MyBioSource (San Diego, CA, USA). Collagenase (MMP-1) was obtained from Sino Biological (Beijing, China). SYN^®^-AKE (dipeptide diaminobutyroyl benzylamide) was purchased from Cayman Chemical (Ann Arbor, MI, USA), and all other peptides were synthesized by and purchased from CUSABIO (Houston, TX, USA). Medimin A (INCI: Methoxy PEG-12 Retinamide) was obtained from HBwell (Icheon, Republic of Korea), and retinol and retinyl palmitate were purchased from KPT (Cheongju, Republic of Korea). The test material Medipep-6PN (INCI name: Palmitoyl Hexapeptide-102) was synthesized and supplied by SKINMED (Daejeon, Republic of Korea). Phosphate-buffered saline (PBS), Tween 20, polyethylene glycol (average molecular weight 8000), LB agar, and bovine serum albumin (BSA) were obtained from Sigma-Aldrich (St. Louis, MO, USA). Other reagents and cell culture media were purchased from WELGENE (Gyeongsan, Republic of Korea). In this study, peptide–target interaction experiments were mainly conducted in the micromolar concentration range, whereas cell-based efficacy assays for cosmetic application were designed at ppm or *w*/*w*% levels to reflect actual formulation concentrations.

### 4.2. Cell Culture

TE671 rhabdomyosarcoma cells used in this study were purchased from the Korean Cell Line Bank (KCLB), and HS68 human dermal fibroblasts were purchased from the American Type Culture Collection (ATCC). The culture medium used was Dulbecco’s Modified Eagle’s Medium (DMEM) supplemented with 10% fetal bovine serum (FBS) and 1% penicillin–streptomycin.

### 4.3. Biopanning

**Figure 9:**
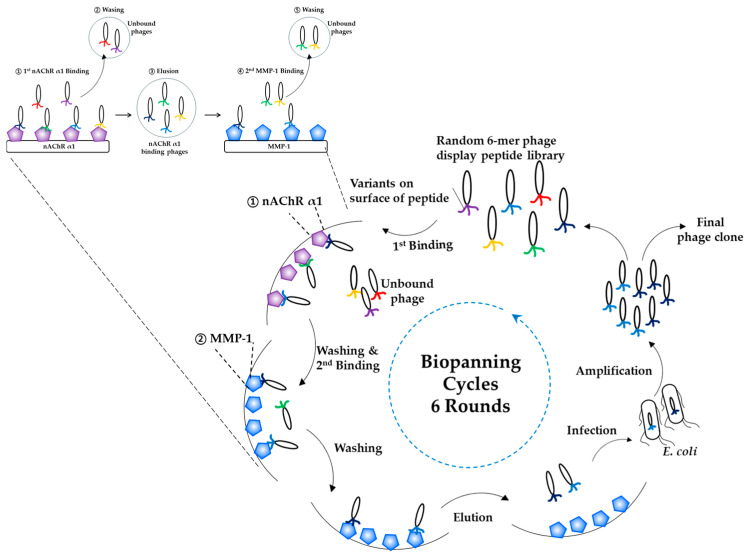
A schematic diagram of muscle AChR α1 and MMP-1-binding peptide screening via phage display-based biopanning. A random 6-mer phage display peptide library was used for sequential biopanning against the two target proteins, muscle nAChR α1 and MMP-1. In the first step, the phage display peptide library was incubated with muscle nAChR α1 (purple pentagons) and washed to remove unbound phages, and the bound phages were then eluted. In the second step, the eluted phages were incubated with MMP-1 (blue pentagons), washed to remove nonspecifically bound phages, and the specifically bound phages were eluted, amplified in *E. coli*, and used in the next round of biopanning.

In this study, biopanning was performed to identify peptides binding to the two target proteins, muscle nAChR α1 and MMP-1, using a random 6-mer phage display peptide library (1.23 × 10^7^ diversity) constructed in a previous study [31] (Figure 9). The target proteins, muscle nAChR α1 and MMP-1, were each diluted in PBS to a concentration of 10 μg/mL and dispensed into 96-well high-binding plates, followed by immobilization at 4 °C overnight. The wells were then blocked with PBS containing 2% BSA at room temperature for 2 h to minimize nonspecific binding. First, the 6-mer phage library was added to plates coated with muscle nAChR α1 and incubated at 30 °C for 1 h. Unbound phages were removed, and phages bound to muscle nAChR α1 were eluted. The eluted phages were then transferred to plates coated with MMP-1 and incubated again at 30 °C for 1 h. Nonspecifically bound phages were removed through washing with PBS containing 0.05% Tween 20. As the biopanning rounds progressed, the number of washes was gradually increased to 3 times for rounds 1–2, 5 times for rounds 3–4, and 6 times for rounds 5–6. The bound phages were eluted by adding 100 μL of 0.2 M glycine-HCl (pH 2.2) and incubating for 10 min, followed by immediate neutralization with 20 μL of 1 M Tris-HCl buffer (pH 9.0). The eluted phages were added to *E. coli* and infected at 37 °C for 1 h, after which helper phage was added, and the culture was incubated at 37 °C for 18 h. The culture supernatant was collected by centrifugation at 12,000× *g* for 10 min at 4 °C. Polyethylene glycol (PEG) precipitation was performed by adding 20% PEG/2.5 M NaCl solution at one-sixth of the supernatant volume, incubating at 4 °C for 1 h, and centrifuging at 12,000× *g* for 1 h at 4 °C to collect the PEG-precipitated phage pellet. The phage pellet was resuspended in 1000 μL PBS to obtain a sub-library enriched for phages binding to muscle nAChR α1 and MMP-1. This sub-library was used as the input phage for the next round of biopanning, and a total of six rounds were performed. In each round, plaque-forming units (PFUs) of the input and output phages were determined to evaluate the selection efficiency.

### 4.4. Enzyme-Linked Immunosorbent Assay (ELISA)

After the sixth round of biopanning, 270 individual phage clones were randomly selected from the recovered phage pool, and an ELISA was performed to evaluate their binding signals to each target protein. Nunc MaxiSorp 96-well plates were coated with muscle nAChR α1 and MMP-1 at 5 μg/mL at 4 °C overnight and then blocked at room temperature with PBS containing 2% BSA. Next, the plates were washed three times with PBS containing 0.05% Tween 20 (PBS-T). After, the culture supernatants of each phage clone were added to the plates and incubated at 37 °C for 1 h, followed by three washes with PBS-T. HRP-conjugated anti-M13 antibody diluted 1:1000 in BSA solution was then added to each well and incubated for 1 h at room temperature, and the plates were washed three times with PBS-T to remove nonspecifically bound antibodies. Subsequently, 3,3′,5,5′-tetramethylbenzidine (TMB) substrate was added to develop color, and the reaction was stopped by adding 2 M H_2_SO_4_. Finally, absorbance at 450 nm was measured using a microplate reader. Positive phage clones were identified by DNA sequencing using the phagemid primer 5′-GATTACGCCAAGCTTTGGAGC-3′ (Bioneer, Daejeon, Republic of Korea).

### 4.5. In Silico Docking Molecular Modeling

In silico docking molecular modeling was performed using LucyNet™ (Pharminogen Inc., Yongin, Republic of Korea), an AI-based drug discovery platform. The three-dimensional structure of human adult muscle-type nicotinic acetylcholine receptor (nAChR; (α1)_2_β1δε) was predicted by homology modeling using the MODELER (SALIGN) module in BIOVIA Discovery Studio 2024. Multi-template modeling was conducted using the bovine adult muscle nAChR resting-state structure (PDB ID: 9AVV), the bovine fetal muscle nAChR structure (PDB ID: 9AVU), and the *Torpedo californica* nAChR structure in complex with α-bungarotoxin (PDB ID: 6UWZ). After sequence alignment, loop regions were refined, and the resulting receptor model was used for subsequent docking simulations. To define a plausible docking region for peptide ligands, the orthosteric binding site observed in the α-bungarotoxin-bound nAChR structure (PDB ID: 6UWZ) was referenced. The peptide-binding pocket was set around the loop C region of the α1 subunit. For ligand preparation, Medipep-6PN (palmitoyl-hexapeptide) and hexapeptide were modeled in a water environment to obtain stable 3D conformations by analyzing structural changes over time. To mimic physiological conditions, Na^+^ and Cl^−^ ions were added to reflect ionic effects relevant to aqueous biological environments. The stabilized peptide conformations were used for docking. For docking, the CHARMm-based docking tool CDOCKER was used. This method places ligands into a specified receptor site, generates diverse poses through random rotations, and searches for energetically favorable binding conformations by applying a simulated molecular dynamics protocol, thereby deriving an optimal binding model based on ligand–receptor interactions. To select the most appropriate pose among the generated docking results, the LigScore2 scoring function was applied. LigScore2 estimates ligand–receptor binding stability using parameters including van der Waals interactions, polar surface terms, and a desolvation penalty term. The LigScore2 scores indicate predicted binding affinity. Final interactions were visualized as hydrogen-bonding networks and hydrophobic interaction maps using Discovery Studio Visualizer.

### 4.6. Surface Plasmon Resonance Assay

The binding properties of the peptides (binding response and binding affinity) were evaluated by SPR analysis using a Biacore T200 instrument (Cytiva, Uppsala, Sweden). A CM5 sensor chip (Series S Sensor Chip CM5, BR100530, Cytiva, Uppsala, Sweden) was activated with an Amine Coupling Kit (BR100050, Cytiva, Uppsala, Sweden), after which muscle nAChR α1 and MMP-1 proteins diluted in running buffer (HBS-EP, BR100669, Cytiva, Uppsala, Sweden) to 5 μg/mL were injected at a flow rate of 10 μL/min and immobilized on the surface of the CM5 sensor chip. To examine binding responses, each of the 11 candidate peptides was diluted to 100 μM in running buffer and injected at a flow rate of 30 μL/min, and the response units (RUs) generated by interactions with immobilized muscle nAChR α1 and MMP-1 were recorded. For kinetic analysis, hexapeptide was diluted in running buffer to 7.8125, 15.625, 31.25, 62.5, 125, 250, 500, 1000, 2000, and 4000 μM and injected at a flow rate of 30 μL/min, and its association and dissociation with muscle nAChR α1 were measured. Palmitoyl-hexapeptide was diluted in running buffer to 0.9765, 1.9531, 3.9062, 7.8125, and 15.625 μM and injected at the same flow rate. For analysis of the interaction with MMP-1, hexapeptide was diluted in running buffer to 2.5, 5, 10, and 20 μM, and palmitoyl-hexapeptide was diluted to 0, 0.25, 0.5, 1, and 2 μM and injected at 30 μL/min. The association rate constant (*k_a_*) and dissociation rate constant (*k_d_*), which describe the kinetics of binding and dissociation, and the equilibrium dissociation constant (*K_D_*) were calculated by fitting the sensorgrams using a 1:1 binding kinetics model in Biacore T200 Evaluation Software version 3.2. When *k_a_* and *k_d_* could not be stably fitted, *K_D_* was determined using a steady-state model.

### 4.7. Ratiometric Fluorescence Imaging

The effect of the peptide on muscle nAChR-mediated intracellular calcium signaling was evaluated by ratiometric fluorescence imaging. TE671 cells expressing muscle nAChR were cultured in DMEM supplemented with 10% FBS and 1% penicillin–streptomycin in a humidified incubator at 37 °C with 5% CO_2_. Coverslips (18 mm diameter) were placed in 12-well culture plates, and cells were seeded at a density of 2 × 10^4^ cells/well (1 mL per well). Cells were allowed to attach to the coverslips for 3 days. For calcium imaging, the coverslips were transferred to new 12-well plates and loaded with Fura-2-AM (Invitrogen, Carlsbad, CA, USA) by adding 3 μL of Fura-2-AM to 997 μL of Hank’s balanced salt solution (HBSS) and incubating the cells at 37 °C for 15 min. After dye loading, cells were washed three times with HBSS to remove residual Fura-2-AM, and 1 mL of HBSS was added to each well. The dye-loaded coverslip was mounted in a chamber (Live Cell Instrument, Namyangju, Republic of Korea), and 500 μL of HBSS was added to the chamber. Cells were pretreated with Medipep-6PN at 0.3, 1, 3, 10, and 30 μM, and the nicotine concentration in the chamber was fixed at 200 μM. Fluorescence signals from 10 to 20 randomly selected cells were recorded using a fluorescence microscope (DMI3000 B, Leica Microsystems, Wetzlar, Germany) with alternating excitation at 340 and 380 nm at 500 ms intervals. The fluorescence ratio (F_340_/F_380_) was calculated using LAS X software (v3.7.4.23463) [59].

### 4.8. Cytotoxicity

The cytotoxicity of Medipep-6PN was assessed according to cell viability using the MTT [3-(4,5-dimethylthiazol-2-yl)-2,5-diphenyltetrazolium bromide] assay. Briefly, HDFs (1 × 10^5^ cells/well) were seeded onto 24-well culture plates and incubated for 18 h. After removing the existing medium, the cells were treated with various concentrations of the test samples diluted in serum-free medium for 24 h. Then, the cells were incubated with 1 μg/mL of MTT solution for 3 h at 37 °C. The resulting formazan crystals were dissolved in 100 μL of DMSO, and the absorbance was measured at 540 nm using a microplate reader. Since the amount of formazan generated is proportional to the absorbance value, the cell viability (%) of each treatment group was calculated based on the untreated control group using the measured absorbance value.

### 4.9. Measurement of Collagenase (MMP-1) Activity Assay

The inhibition of collagenase (MMP-1) was evaluated using the EnzChek Gelatinase/Collagenase assay kit (Cat No. E12055, Invitrogen, Carlsbad, CA, USA). The total reaction volume was set to 200 μL, and black 96-well plates were used to block light. Then, 110 μL of reaction buffer was added to each well of the 96-well plate, followed by the addition of 20 μL of test substance or 20 μL of the positive control, 1,10-phenanthroline monohydrate (10 mM). The plate was shaken 2–3 times. Subsequently, 20 μL of the enzyme (10 U/mL) and 50 μL of the substrate, collagen type I (100 μg/mL), were added, and the mixture was incubated for 1 h at 37 °C. After the reaction, fluorescence was measured at 495/515 nm using a fluorescence microplate reader. The collagenase activity inhibition rate was calculated according to the following formula:

Inhibition rate (%) = (1 − (F_sample_ − F_blank_)/(F_control_ − F_blank_)) × 100

where F_sample_ is the fluorescence value of the sample-treated group (enzyme + substrate + sample), F_control_ is the fluorescence value of the control group treated with the enzyme and substrate only (no sample), and F_blank_ is the fluorescence value of the blank group containing the substrate only without the enzyme. IC_50_ values were calculated in GraphPad Prism 10 (GraphPad Software, San Diego, CA, USA) by nonlinear regression using a four-parameter logistic model ([inhibitor] vs. response, variable slope).

### 4.10. Measurement of Collagen Synthesis

HDFs were seeded a density of 2 to 5 × 10^4^ cells per well in a 48-well culture plates and incubated in DMEM containing 5% FBS and 1% penicillin–streptomycin for 24 h. After removing the existing medium, fresh medium containing the samples was added. After an additional 24 h of incubation, the cell culture supernatant was collected for ELISA analysis. The Human procollagen Iα1 DuoSet^®^ ELISA kit (DY6220-05, R&D Systems, Minneapolis, MN, USA) was used, and the evaluation was performed according to the manufacturer’s provided experimental protocol.

### 4.11. Measurement of UVB-Induced Collagenase (MMP-1) Gene Expression

The inhibitory effect on UVB-induced MMP-1 gene expression was evaluated using reverse-transcription polymerase chain reaction (RT-PCR). HDFs were seeded into 6-well plates and allowed to stabilize. After stabilization, the cells were washed with PBS and irradiated with UVB at 10 mJ/cm^2^. Immediately after UVB irradiation, fresh medium containing the test samples was added, and the cells were incubated for 24 h. The samples were diluted in the cell culture medium to the target concentrations before treatment. Total RNA was extracted from the cells using Tri Reagent (Molecular Research Center, Cincinnati, OH, USA), and the concentration and purity of RNA were measured with a NanoDrop spectrophotometer (Thermo Fisher Scientific, Waltham, MA, USA). Using the extracted total RNA (1 μg) as a template, complementary DNA (cDNA) was synthesized in a 10 μL reaction volume with oligo(dT) primers and M-MLV reverse transcriptase (Promega, Madison, WI, USA). The synthesized cDNA was then used as a template for PCR with gene-specific primers (Table 5), and the amplified products were subjected to electrophoresis on 1% agarose gel. Band intensity was quantified using ImageJ software v1.54r (National Institutes of Health, Bethesda, MD, USA) and changes in MMP-1 gene expression were analyzed. The primer sequences used for gene expression analysis are shown in Table 5.

### 4.12. Immunocytochemistry (ICC)

The expression of procollagen Iα1 in HDFs was confirmed using immunofluorescence staining. HDFs (1 × 10^4^ cells/well) were seeded onto 12-well culture plates containing sterilized coverslips (22 × 22 mm) and pre-incubated for 24 h. The cells were then washed with PBS, and a cream formulation containing Medipep-6PN was diluted in serum-free DMEM to final Medipep-6PN concentrations of 0.1 and 1 ppm, followed by incubation for 24 h at 37 °C in 5% CO_2_. After treatment, the cells were washed twice with PBS and fixed with 4% paraformaldehyde solution for 10 min at room temperature. The fixed cells were washed twice with PBS and then permeabilized with 0.2% Triton X-100 for 5 min. To prevent nonspecific antibody binding, the cells were blocked with 1% BSA solution for 60 min at room temperature. After washing twice with Tris-buffered saline (TBS) containing 0.1% Triton X-100, the cells were incubated with a procollagen Iα1 primary antibody (1:200; Santa Cruz (sc-293182), Dallas, TX, USA) overnight at 4 °C. After washing twice with TBS, the cells were treated with Alexa Fluor 488 goat anti-mouse IgG secondary antibody (1:1000; Thermo Fisher Scientific (A11029), Waltham, MA, USA) for 1 h at room temperature. Finally, after washing twice with TBS and mounting with Vectashield anti-fade mounting medium containing DAPI (Vector Laboratories Inc (H-1200), Newark, CA, USA), fluorescence images were acquired using a Leica TCS SP8 confocal microscope (Leica Microsystems, Wetzlar, Germany).

### 4.13. Clinical Study

The clinical study was conducted as a randomized, double-blind, split-face study in 25 Asian adults aged 30 to 65 years with wrinkles around the eyes. Before the start of the study, written informed consent was obtained from all participants. This study was approved by the Institutional Review Board of SKINMED Co., Ltd. (Approval Number: SKINMEDIRB-SM02-25F24-098) on 20 February 2025, and all procedures were conducted in accordance with the Declaration of Helsinki of the World Medical Association for research involving human subjects. Individuals who met the exclusion criteria, such as having used products with wrinkle-improving effects on the test area within 3 months prior to the start of the study or having undergone cosmetic procedures on the test area within 6 months, were excluded from participation. To ensure the objectivity of randomization, participants were allocated to one of five test groups (Medipep-6PN, SYN^®^-AKE, Medimin A, retinol, or retinyl palmitate; five per group) according to a computer-generated randomization table. For allocation concealment, independent personnel not involved in the clinical evaluation prepared the coded products, and complete blinding was maintained by providing the test substance and placebo in identical containers. Additionally, following a split-face design, the side of the face (left or right) to which the test substance was applied was randomly determined, and measures were taken to ensure that participants, researchers, and outcome assessors remained unaware of the allocation information until the analysis was completed. The concentrations of the test materials were set on the basis of manufacturer-recommended use levels, previous human application studies, and regulatory guidelines. The concentration of Medipep-6PN was set at 500 ppm, which had been confirmed as safe in a human skin irritation test, and the concentration of SYN^®^-AKE followed the manufacturer’s recommended use concentration [60,61]. The concentrations of Medimin A (0.2%), retinol (2500 IU/g), and retinyl palmitate (10,000 IU/g) were established with reference to the maximum allowable levels presented in the regulations on functional anti-wrinkle cosmetics of the Ministry of Food and Drug Safety (MFDS), Republic of Korea [62]. These levels are also generally consistent with the concentration ranges used in previous human application studies of Medimin A and retinoids [63,64,65]. All subjects applied the assigned test product once daily in the evening for 4 weeks, applying the designated test product to one side of the eye area and a placebo to the contralateral side. All evaluations were conducted at the SKINMED Clinical Trials Center under a controlled indoor environment (temperature 20–24 °C, relative humidity 40–60%) according to the center’s standard operating procedures (SOPs). Wrinkle-improving efficacy was assessed at baseline (week 0) and after 4 weeks of use (week 4) using a three-dimensional skin imaging device (Antera 3D^®^, Miravex Ltd., Dublin, Ireland), which combines skin profilometry, multispectral analysis, and colorimetry to reconstruct the skin surface in three dimensions and perform subsequent image analysis. Images of the crow’s feet area on both sides of the face were acquired at baseline and at week 4, and wrinkle depth and volume were quantified on each side for intra-individual comparison.

Skin safety and tolerability were assessed through visual inspection by a board-certified dermatologist (or trained researcher) and subjective self-reporting by the subjects. Adverse events were monitored at each visit (baseline, week 2, and week 4) based on the guidelines of the International Contact Dermatitis Research Group (ICDRG) and Frosch & Kligman. Objective signs such as erythema, edema, and scaling were graded on a 5-point scale (0 = none to 4 = severe). Additionally, subjects were instructed to record any subjective sensations of irritation, including itching, stinging, or burning, in a daily symptom diary throughout the study period. The investigator reviewed these records to determine the presence and severity of any adverse skin reactions.

### 4.14. Statistical Analysis

Statistical analyses were performed using SPSS software (version 26.0; IBM Corp., Armonk, NY, USA) and GraphPad Prism (version 10.0; GraphPad Software, San Diego, CA, USA). The significance level (α) was set at 0.05. For in vitro data, statistical significance was determined using several methods. One-way analysis of variance (ANOVA) followed by Tukey’s multiple comparisons test was used for comparisons across multiple treatment groups, while two-way ANOVA followed by Sidak’s multiple comparisons test was employed for experiments involving two independent variables. For clinical data, the Shapiro–Wilk test was used to assess the normality of the data. For variables that satisfied the normality assumption (*p* > 0.05), a parametric repeated-measures ANOVA was applied. For variables that did not meet the normality criteria (*p* ≤ 0.05), the nonparametric Friedman test and Wilcoxon signed-rank test were used. *p*-values were adjusted using Bonferroni correction to account for multiple comparisons. Based on these analyses, differences were considered statistically significant at an adjusted *p*-value of <0.025.

## 5. Conclusions

In this study, we used phage display to discover Medipep-6PN, a novel hexapeptide that targets muscle nAChR α1 and MMP-1, and confirmed in vitro that it regulates muscle nAChR-mediated intracellular calcium signaling, inhibits MMP-1 activity, and MMP-1 mRNA expression, and increases type I collagen biosynthesis.

This study elucidated the inhibitory mechanisms of Medipep-6PN, a peptide–lipid conjugate, against muscle nAChR and MMP-1, and explored its potential as an anti-wrinkle agent through a small-scale pilot clinical trial. While Medipep-6PN demonstrated improvements in crow’s feet wrinkle parameters and an excellent safety profile, this study has limitations regarding the small clinical sample size and wide age distribution of participants. Therefore, although our findings suggest that Medipep-6PN is a promising anti-aging candidate, definitive establishment of its efficacy warrants further confirmation through large-scale, confirmatory clinical trials.

## Figures and Tables

**Table 1:** Summary table of O/I phage ratio results by biopanning round.

Round	Input Phages (PFU/mL)	Output Phages (PFU/mL)	Output/Input Phage Ratio	Fold Increase
1	8.00 × 10^11^	1.90 × 10^7^	2.37 × 10^−5^	1
2	2.44 × 10^12^	1.14 × 10^8^	4.67 × 10^−5^	1.9
3	4.80 × 10^13^	3.53 × 10^9^	7.35 × 10^−5^	3.1
4	7.20 × 10^13^	2.63 × 10^10^	3.65 × 10^−4^	15.4
5	1.76 × 10^13^	1.82 × 10^10^	1.03 × 10^−3^	43.4
6	2.26 × 10^13^	4.00 × 10^10^	1.77 × 10^−3^	74.6

**Table 2:** Information about the 11 candidate peptides selected through biopanning.

No.	Clone Number	Sequence	Muscle nAChR Subunit α1 /BSA Ratio	MMP-1 /BSA Ratio
1	#11	RKWRYR	4.01	3.48
2	#68	KKQRVK	3.40	2.39
3	#83	KRQSER	2.67	2.68
4	#96	KRQRSK	2.61	2.58
5	#124	RRRQLR	1.57	5.40
6	#128	KKGGAR	1.80	6.14
7	#129	KKQRGK	1.98	5.89
8	#185	KRLRWK	1.89	4.44
9	#210	KRRSQK	1.78	6.04
10	#225	RRQRSR	1.87	5.16
11	#256	KRQPVR	4.49	4.58

**Table 3:** Summary of equilibrium dissociation constant (*K_D_*) and chi-square (Chi^2^) goodness-of-fit values. Data were analyzed using Biacore T200 evaluation software.

Ligand	Analyte	*k_a_* (M^−1^ s^−1^)	*k_d_* (s^−1^)	Rmax (RU)	*K_D_* (M)	Chi^2^	U-Value	Evaluation Model
nAChR α1	Hexapeptide	-	-	463	2.52 × 10^−3^	5.29	-	Steady state
	Palmitoyl-hexapeptide	5.38 × 10^3^	5.14 × 10^−2^	34.69	9.56 × 10^−6^	0.698	4	Kinetics
MMP-1	Hexapeptide	1.06 × 10^3^	6.66 × 10^−3^	18.68	6.24 × 10^−6^	2.64	7	Kinetics
	Palmitoyl-hexapeptide	7.70 × 10^3^	9.62 × 10^−3^	51.69	1.25 × 10^−6^	1.14	2	Kinetics

*k_a_*: association rate constant; *k_d_*: dissociation rate constant; Rmax: maximum binding capacity; *K_D_*: equilibrium dissociation constant; -: not applicable for steady-state fitting; Chi^2^: squared difference between experimental data and fitted curve; U-Value: uniqueness value.

**Table 4:** Summary of results for changes in wrinkle depth and volume before and after application of each substance to evaluate improvement in crow’s feet wrinkles.

Test Group	Test Product	Parameter	Week 0 (Mean ± SD)	Week 4 (Mean ± SD)	Improvement (%)
a	Control	Depth	0.0408 ± 0.0061	0.0402 ± 0.0059	1.47
		Volume	0.4822 ± 0.1567	0.4626 ± 0.1529	4.06
	Medipep-6PN	Depth	0.0374 ± 0.0018	0.0336 ± 0.0016	10.16 *
		Volume	0.4308 ± 0.1998	0.3748 ± 0.1764	13.00 *
b	Control	Depth	0.0468 ± 0.0006	0.0462 ± 0.0006	1.28
		Volume	0.7266 ± 0.1749	0.7038 ± 0.1823	3.14
	SYN^®^-AKE	Depth	0.0440 ± 0.0035	0.0406 ± 0.0054	7.73 *
		Volume	0.764 ± 0.3842	0.7166 ± 0.3841	6.20 *
c	Control	Depth	0.0504 ± 0.010	0.0482 ± 0.0099	4.37
		Volume	0.4584 ± 0.0642	0.4478 ± 0.0803	2.31
	Medimin A	Depth	0.0418 ± 0.0052	0.0354 ± 0.0063	15.31 *
		Volume	0.4222 ± 0.0596	0.3148 ± 0.0788	25.44 *
d	Control	Depth	0.0514 ± 0.0084	0.0512 ± 0.0094	0.39
		Volume	0.5552 ± 0.0591	0.5528 ± 0.0680	0.43
	Retinol	Depth	0.0568 ± 0.0127	0.0510 ± 0.0142	10.21 *
		Volume	0.7018 ± 0.1090	0.5916 ± 0.1515	15.70 *
e	Control	Depth	0.0476 ± 0.0081	0.0454 ± 0.0064	4.62
		Volume	0.6152 ± 0.1038	0.6376 ± 0.1245	−3.64
	Retinyl palmitate	Depth	0.0482 ± 0.0089	0.0408 ± 0.0057	15.35 *
		Volume	0.3852 ± 0.2540	0.3218 ± 0.2147	16.46 *

* *p* < 0.05 vs. baseline (significance before and after application compared to week 0). All test groups (Medipep-6PN, SYN^®^-AKE, Medimin A, retinol, retinyl palmitate) demonstrated statistically significant wrinkle improvement compared to baseline at week 4.

**Table 5:** The primer sequences for gene expression analysis.

Target Gene	Forward Primer (5′→3′)	Reverse Primer (5′→3′)
GAPDH	GCA CCA CCA ACT GCT TAG C	CTT GAT GAC CTC AAC TAC G
MMP-1	TGT TTT GGA AAC TCT CAG GAA	TCA GGG TCA CTC TCG TCA C

## Data Availability

The original contributions presented in this study are included in the article/Supplementary Materials. Further inquiries can be directed at the corresponding author(s).

## Supplementary Materials

The following supporting information can be downloaded at: https://www.mdpi.com/article/10.3390/ijms27041753/s1.

## Appendix A

**Table A1:** Mass spectrometry and chromatography data of the synthesized peptides are provided in the appendix.

No	Peptide Number	Sequence	Lot Number
1	#11	RKWRYR	N0425P9
2	#68	KKQRVK	N0411P5
3	#83	KRQSER	N0408P15
4	#96	KRQRSK	N0408P16
5	#124	RRRQLR	N0408P11
6	#128	KKGGAR	N0416P1
7	#129	KKQRGK	N0416P2
8	#185	KRLRWK	N0408P14
9	#210	KRRSQK	N0408P10
10	#225	RRQRSR	N0408P12
11	#256	KRQPVR	N0408P13
