# Supplementary material for: In Vitro and Clinical Evaluation of the Anti-Wrinkle Efficacy of Medipep-6PN, a Novel Peptide Identified by Phage Display
Source: Int J Mol Sci. 2026 Feb 11;27(4):1753. doi: 10.3390/ijms27041753 (PMC12940413; doi:10.3390/ijms27041753)
Supplement: Supplementary file 1 [file ijms-27-01753-s001.zip › ijms-4090889-supplementary.pdf]

Supplementary Materials

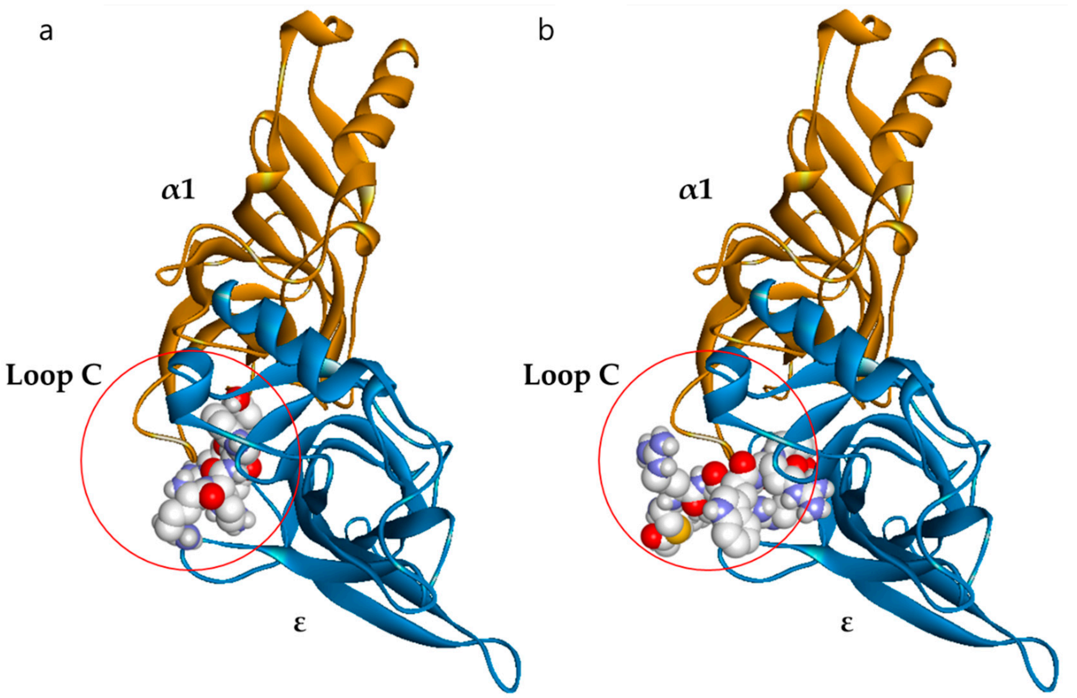

Supplementary Figure S1. Predicted binding poses of hexapeptide and palmitoyl-hexapeptide at loop C region of muscle nAChR α1. (a) Predicted binding pose of hexapeptide at interface between muscle nAChR α1 and ε subunit near loop C. (b) Predicted binding pose of palmitoyl-hexapeptide at same binding pocket.

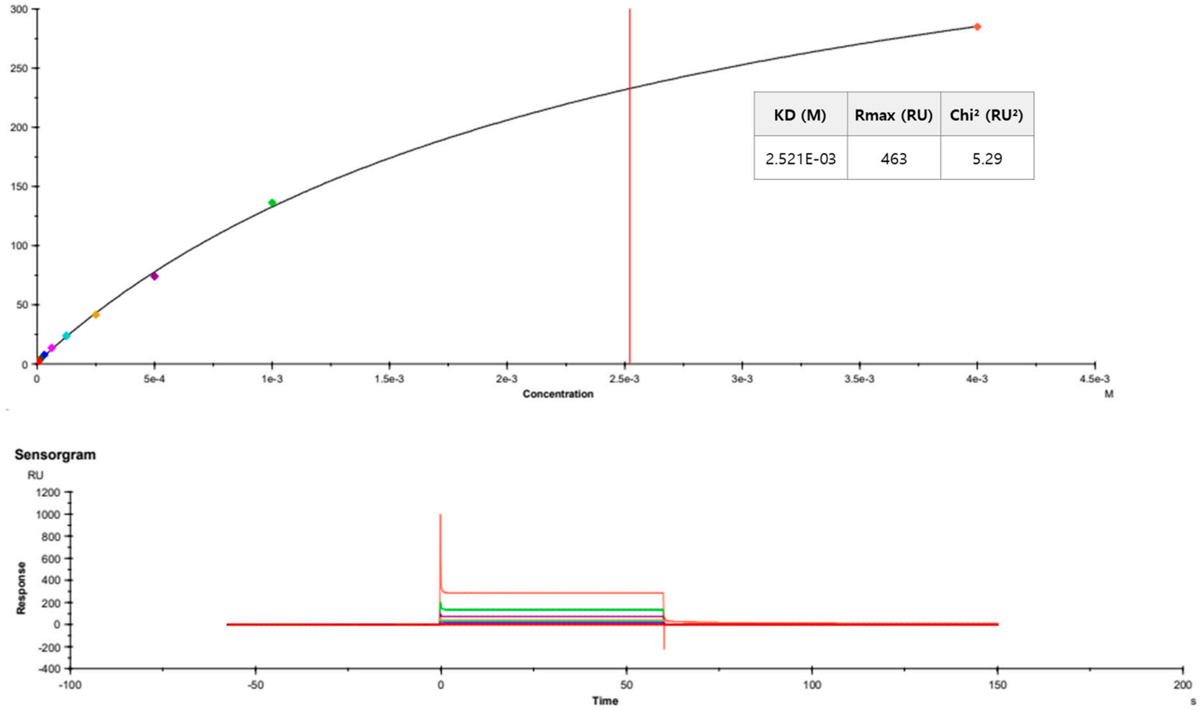

Supplementary Figure S2. SPR sensorgram and steady-state binding curve of hexapeptide binding to muscle nAChR α1.

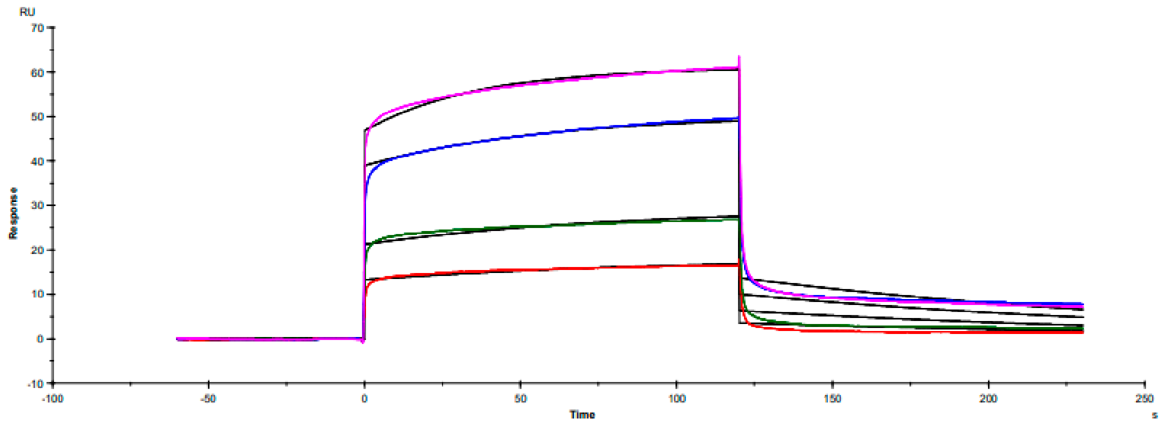

Supplementary Figure S3. SPR sensorgram of hexapeptide binding to MMP-1.

Supplementary Table S1. The sequencing results of the screened peptides.

No	Sample Name	Primer	result	Data Group	DNA Sequence
1	#11	phagemidprimer	GOOD	S	GCAGCACAACTAGCAAGAAAATGGAGATACAGAGCAGCAGCAGAACAAAAACTAATAAGCGAAGAAGACCTAAGCAGATAGGACGACGACGAC
2	#68	phagemidprimer	GOOD	S	GCAGCACAACTAGCAAAAAAACAAAGAGTAAAAGCAGCAGCAGAACAAAAACTAATAAGCGAAGAAGACCTAAGCAGATAGGACGACGACGAC
3	#83	phagemidprimer	GOOD	S	GCAGCACAACTAGCAAAAAGACAAAGCGAAAGAGCAGCAGCAGAACAAAAACTAATAAGCGAAGAAGACCTAAGCAGATAGGACGACGACGAC
4	#96	phagemidprimer	GOOD	S	GCAGCACAACTAGCAAAAAGACAAAGAAGCAAAGCAGCAGCAGAACAAAAACTAATAAGCGAAGAAGACCTAAGCAGATAGGACGACGACGAC
5	#124	phagemidprimer	GOOD	S	GCAGCACAACTAGCAAGAAGAAGACAACTAAGAGCAGCAGCAGAACAAAAACTAATAAGCGAAGAAGACCTAAGCAGATAGGACGACGACGAC
6	#128	phagemidprimer	GOOD	S	GCAGCACAACTAGCAAAAAAAGGAGGAGCAAGAGCAGCAGCAGAACAAAAACTAATAAGCGAAGAAGACCTAAGCAGATAGGACGACGACGAC
7	#129	phagemidprimer	GOOD	S	GCAGCACAACTAGCAAAAAAACAAAGAGGAAAAGCAGCAGCAGAACAAAAACTAATAAGCGAAGAAGACCTAAGCAGATAGGACGACGACGAC
8	#185	phagemidprimer	GOOD	S	GCAGCACAACTAGCAAAAAGACTAAGATGGAAAGCAGCAGCAGAACAAAAACTAATAAGCGAAGAAGACCTAAGCAGATAGGACGACGACGAC
9	#210	phagemidprimer	GOOD	S	GCAGCACAACTAGCAAAAAGAAGAAGCCAAAAAGCAGCAGCAGAACAAAAACTAATAAGCGAAGAAGACCTAAGCAGATAGGACGACGACGAC
10	#225	phagemidprimer	GOOD	S	GCAGCACAACTAGCAAGAAGACAAAGAAGCAGAGCAGCAGCAGAACAAAAACTAATAAGCGAAGAAGACCTAAGCAGATAGGACGACGACGAC
11	#256	phagemidprimer	GOOD	S	GCAGCACAACTAGCAAAAAGACAACCAGTAAGAGCAGCAGCAGAACAAAAACTAATAAGCGAAGAAGACCTAAGCAGATAGGACGACGACGAC

Supplementary Table S2. Skin side effect results of five products in clinical study.

Test product	Adverse Reactions	After Application	After 4 Weeks
Medipep-6PN	No Symptoms	5 (100.0%)	5 (100.0%)
	Erythema	0 (0.0%)	0 (0.0%)
	Itching	0 (0.0%)	0 (0.0%)
	Stinging	0 (0.0%)	0 (0.0%)
Syn®-Ake	No Symptoms	5 (100.0%)	5 (100.0%)
	Erythema	0 (0.0%)	0 (0.0%)
	Itching	0 (0.0%)	0 (0.0%)
	Stinging	0 (0.0%)	0 (0.0%)
Medimin A	No Symptoms	5 (100.0%)	5 (100.0%)
	Erythema	0 (0.0%)	0 (0.0%)
	Itching	0 (0.0%)	0 (0.0%)
	Stinging	0 (0.0%)	0 (0.0%)
Retinol	No Symptoms	5 (100.0%)	5 (100.0%)
	Erythema	0 (0.0%)	0 (0.0%)
	Itching	0 (0.0%)	0 (0.0%)
	Stinging	0 (0.0%)	0 (0.0%)
Retinyl palmitate	No Symptoms	5 (100.0%)	5 (100.0%)
	Erythema	0 (0.0%)	0 (0.0%)
	Itching	0 (0.0%)	0 (0.0%)
	Stinging	0 (0.0%)	0 (0.0%)
